# Transcriptome analysis reveals candidate genes involved in luciferin metabolism in *Luciola aquatilis* (Coleoptera: Lampyridae)

**DOI:** 10.7717/peerj.2534

**Published:** 2016-10-04

**Authors:** Wanwipa Vongsangnak, Pramote Chumnanpuen, Ajaraporn Sriboonlert

**Affiliations:** 1 Department of Zoology, Kasetsart University, Bangkok, Thailand; 2 Computational Biomodelling Laboratory for Agricultural Science and Technology (CBLAST), Faculty of Science, Kasetsart University, Bangkok, Thailand; 3 Department of Genetics, Kasetsart University, Bangkok, Thailand; 4 Centre for Advanced Studies in Tropical Natural Resources, Kasetsart University, Bangkok, Thailand

**Keywords:** Firefly bioluminescence, Functional annotation, Luciferase, RNA-seq

## Abstract

Bioluminescence, which living organisms such as fireflies emit light, has been studied extensively for over half a century. This intriguing reaction, having its origins in nature where glowing insects can signal things such as attraction or defense, is now widely used in biotechnology with applications of bioluminescence and chemiluminescence. Luciferase, a key enzyme in this reaction, has been well characterized; however, the enzymes involved in the biosynthetic pathway of its substrate, luciferin, remains unsolved at present. To elucidate the luciferin metabolism, we performed a *de novo* transcriptome analysis using larvae of the firefly species, *Luciola aquatilis*. Here, a comparative analysis is performed with the model coleopteran insect *Tribolium casteneum* to elucidate the metabolic pathways in *L. aquatilis*. Based on a template luciferin biosynthetic pathway, combined with a range of protein and pathway databases, and various prediction tools for functional annotation, the candidate genes, enzymes, and biochemical reactions involved in luciferin metabolism are proposed for *L. aquatilis*. The candidate gene expression is validated in the adult *L. aquatilis* using reverse transcription PCR (RT-PCR). This study provides useful information on the bio-production of luciferin in the firefly and will benefit to future applications of the valuable firefly bioluminescence system.

## Introduction

The firefly is a bioluminescent beetle belonging to the Order Coleoptera, Family Lampyridae. Over 100 genera and 2,000 species of fireflies have been reported around the world both in temperate and tropical areas (McDermott, 1964; McDermott, 1966; Branham, 2010). Of these 100 genera, *Photinus* and *Photuris* from North America (Lewis & Cratsley, 2008; Faust, De Cock & Lewis, 2012; Stansbury & Moczek, 2014; Martin et al., 2015; Sander & Hall, 2015) and *Pyrocoelia* (Fu et al., 2006a) and *Luciola* (Tsutomu, Hiroki & Eiichi, 1989; Fu et al., 2006b; Oba et al., 2006; Ohtsuki et al., 2008; Oba & Kainuma, 2009; Oba et al., 2010) from Asia are the most studied, particularly their behaviors and cellular mechanisms. Bioluminescence, regarded as the most striking characteristic of fireflies, is a property generated by a chemical reaction in the photocyte cells situated in the sixth and seventh ventral segments of fireflies (Greenfield, 2001; Stanger-Hall, Lloyd & Hillis, 2007; Goh & Li, 2011). Firefly bioluminescence is catalyzed by a luciferase enzyme in the presence of O_2_, ATP, and Mg^2+^ (Deluca, 1976; Baldwin, 1996) in a two-step reaction; D-luciferin is adenylated by ATP at the luciferase active site and converted into luciferyl-adenosine monophosphate (luciferyl-AMP). This luciferyl-AMP is then oxidized and converted into excited state oxyluciferin. This excited state oxyluciferin later returns to its ground state by the emission of a visible photon, thereby generating visible light (Fraga, 2008; Naumov et al., 2009; Inouye, 2010; Pinto da Silva, Santos & Esteves da Silva, 2012).

Firefly luciferase, the key enzyme in the firefly bioluminescence reaction, is well characterized. This enzyme was first purified and crystallized in 1956 by Green and McElroy (Green & McElroy, 1956; Fraga, 2008). Later in 1985, it was cloned and expressed in *Escherichia coli* (De Wet et al., 1985). The structure of the luciferase from North American firefly *P. pyralis* was subsequently determined in 1996 (Conti, Franks & Brick, 1996). So far, firefly luciferase has been utilized in various molecular and medical studies. For instance, the firefly luciferase gene is widely used as a reporter gene in gene expression analysis (De Wet et al., 1985; Koncz et al., 1990). Firefly luciferases have been used in different applications, e.g., bioimaging (Calvo-Álvarez et al., 2015; Reimão et al., 2015), protein-protein interaction assay (Kurihara et al., 2016), immunoassay (Smirnova, Samsonova & Ugarova, 2016), and ATP quantification (Marques & Esteves da Silva, 2009). However, knowledge about the biosynthesis of the luciferase substrate, luciferin, is still lacking. To date, only the structure and chemical reactions of luciferin have been characterized (White, McCapra & Field, 1963; Fraga, 2008). The bioluminescence systems used in these applications rely solely on commercially synthesized luciferin. Many attempts to resolve the luciferin biosynthetic pathway have been performed (Okada et al., 1974; Okada, Iio & Goto, 1976; McCapra & Razavi, 1975; McCapra & Razavi, 1976; Colepicolo, Pagni & Bechara, 1988; Niwa, Nakamura & Ohmiya, 2006). Recently, Oba et al. (2013) analyzed the luciferin biosynthetic pathway by injection of isotope-labeled compounds L-cysteine, hydroquinone, and benzoquinone into an adult lantern of firefly *L. lateralis*. Luciferin is demonstrated to be synthesized from 1,4-hydroquinone and two endogenous L-cysteine molecules (Oba et al., 2013). The genes involved in firefly bioluminescence pathway were investigated by Viviani and colleagues in 2013. A complementary DNA (cDNA) library of *Macrolampis* sp2 lantern was constructed and sequenced; however, no gene product could be directly associated with luciferin biosynthesis (Viviani, Carmargo & Amaral, 2013).

For various insect species, transcriptome studies using RNA sequencing to elucidate gene networks involved in many biological pathways, e.g., olfactory mechanisms in moth (Zhang et al., 2015), visual mechanism in dragonfly (Futahashi et al., 2015), and bioluminescence mechanism in glowworm (Sharpe et al., 2015) have been done. Protein coding genes potentially involved in bioluminescent metabolism, including candidate luciferases, were identified in the New Zealand glowworm, *Arachnocampa luminosa* (Diptera) utilizing high-throughput sequencing technology (Sharpe et al., 2015). In Lepidoptera, the olfactory mechanisms from two pest species *Helicoverpa armigera* and *H. assulta* were studied (Zhang et al., 2015). Transcripts isolated from the antenna of the two species were sequenced using Illumina sequencing technology. They identified 133 putative chemosensory unigenes in *H. armigera* and 131 putative chemosensory genes in *H. assulta* (Zhang et al., 2015). Another example of using RNA sequencing on insect transcriptome analysis is the study of color vision opsin genes in dragonflies (Futahashi et al., 2015). This study identified 20 opsin genes in dragonflies of the Family Libellulidae (Futahashi et al., 2015). Recently, transcriptome analyses have been utilized to elucidate the opsin gene evolution in North American fireflies (Martin et al., 2015; Sander & Hall, 2015). Both RNA and genome sequencing were performed using Illumina HiSeq 2000. A total of 172 million reads were obtained from the heads of 10 firefly species. Two opsin genes were identified in their study (Sander & Hall, 2015). However, the other annotated genes derived from their transcriptome data in the study have not yet been reported.

Therefore, our study aims to reveal expressed genes in luciferin metabolism using *de novo* transcriptome analysis from the Illumina RNA sequencing of a Thai native firefly, *L. aquatilis*. Based on the transcriptome data, we used a range of protein and pathway databases combined with prediction tools to annotate the protein coding genes of *L. aquatilis*. Candidate genes involved in the luciferin metabolic pathway were subsequently proposed based on the studies performed by Niwa, Nakamura & Ohmiya (2006); Oba et al. (2013); Hemmati et al. (2015); Kanie et al. (2016). We validated expression of these candidate genes using reverse transcription PCR (RT-PCR) analysis in another developmental stage with the bioluminescent activity, i.e., the adult *L. aquatilis.* The proposed enzymes in this study provides an insight into the cryptic luciferin biosynthesis pathway in the firefly. It is worth to note that gene knockout and expression analysis are required to confirm the proposed functions of these enzymes. Prospectively, elucidation of this pathway will facilitate the development of gene reporter system, live cell imaging and other related technologies in the future.

## Materials and Methods

### Sample collection, RNA isolation, and RNA sequencing

*L. aquatilis* larvae were collected from Nakorn Ratchasrima province, Thailand. Total RNA was extracted from three specimens of bioluminescent *L. aquatilis* larvae. All three specimens were ground to a fine powder in liquid nitrogen with a mortar and pestle. Total RNA was extracted using TRIzol^®^Reagent (Thermo Fisher Scientific, Waltham, MA, USA) according to the manufacturer’s protocol. The total RNA was treated with DNase I (Qiagen, Hilden, Germany) as described in the manufacturer’s protocol. Pooled RNA sample was sent for sequencing at Macrogen (South Korea) using Illumina HiSeq 2000 Sequencing System (Illumina, San Diego, CA, USA). Quality and quantity of RNA were measured using an Agilent 2100 Bioanalyzer (Agilent Technologies, Santa Clara, CA, USA). The mRNA was converted into a library of template molecules using the reagents provided in the Illumina^®^TruSeq™ RNA Sample Preparation Kit (Illumina). The poly-A containing mRNA molecules were purified using poly-T oligo-attached magnetic beads. Following purification, the mRNA is fragmented into small pieces using divalent cations under elevated temperature. The cleaved RNA fragments were converted into first strand cDNA using reverse transcriptase and random primers, followed by second strand cDNA synthesis using DNA Polymerase I and RNase H. These cDNA fragments then went through an end repair process using an End Repair (ERP) mix. A single ‘A’ nucleotide was added to the 3^′^ ends of the blunt fragments to prevent them from ligating to one another during the adapter ligation reaction. Adapters were then added to the ends of the ds cDNA, preparing them for hybridization onto a flow cell. DNA Fragments were enriched by PCR and subsequently sequenced using Illumina HiSeq 2000 Sequencing System (Illumina, USA) which is able to generate paired-end read with 2 × 100 base pairs (bp) read length.

### *De novo* transcriptome assembly

FASTQC (Version 0.11.3) was used to determine the quality of the RNA sequencing data (www.bioinformatics.babraham.ac.uk/projects/fastqc/). Reads were trimmed and cleaned using Trimmomatic (v. 0.32; Bolger, Lohse & Usadel, 2014). Sequences with a quality score equal to or greater than 15 and a minimum length of 36 bp were retained. After raw reads filtering, *de novo* assembly of transcripts data was performed using Trinity RNA-Seq assembly (release 17.07.2014; https://github.com/trinityrnaseq/trinityrnaseq/). The transcript abundance, Fragments Per Kilobase of transcript per Million mapped reads (FPKM; Trapnell et al., 2010), was calculated using Trinity based on RSEM algorithm (Li & Dewey, 2011).

### Gene prediction and functional annotation of *L. aquatilis*

All protein-coding genes were predicted and extracted from assembled transcripts using TransDecoder (https://transdecoder.github.io/). Protein coding genes were identified via TransDecoder under the following criteria: a minimum length of 100 amino acids, a default log-likelihood score of greater than 0 and matches within the Pfam (pfam.xfam.org/) and UniProtKB/Swiss-Prot databases (web.expasy.org/docs/swiss-prot_guideline.html).

To assign protein functions, non-redundant protein (NR), and Uniprot databases were used for functional annotation. The cut-off values for each search were adjusted according to the size of the databases. An annotation with the bigger NR database was performed using a minimum amino acid sequence identity of 50% and E-value of 1E–10. Subsequently, the annotation with the smaller Uniprot database was performed using a minimum amino acid sequence under identity of 25% and E-value of 1E–05. In addition, GhostKOALA (www.kegg.jp/ghostkoala/) and BlastKOALA (www.kegg.jp/blastkoala/) tools in the metabolic pathway database, KEGG, were also used for functional annotation with a 50% identity cutoff. The annotated genes obtained from the KEGG database were categorized based on their functional roles. In addition, the protein-coding genes annotated via TransDecoder were further analyzed using KEGG taxonomic mapping.

### Comparative protein sequence analysis between *L. aquatilis* and closely related species

The result from KEGG taxonomic mapping showed that the majority of the protein-coding genes in *L. aquatilis* matched to genes from various arthropod species. We further selected the most closely related species presented to assist in functional annotation. The pairwise comparison of protein sequences using BLASTP was subsequently performed between *L. aquatilis* and the most closely related species. The criteria for similarity searching were bidirectional best hits (BBH) with E-value of 1E–05 as a cut-off.

### Identification of candidate genes, enzymes, and metabolic pathways associated with luciferin

The candidate luciferin metabolic pathway of *L. aquatilis* was identified based on the template luciferin pathway by Oba et al. (2013) with some modifications from different studies (Niwa, Nakamura & Ohmiya, 2006; Hemmati et al., 2015; Kanie et al., 2016). To identify all possible candidate genes and enzyme functions for assigning into each step of the biochemical reactions in the luciferin metabolic pathway of *L. aquatilis*, protein sequences of *L. aquatilis* obtained from this study were blasted against three different protein and pathway databases, NR, KEGG, and Uniprot using BLASTP. The protein sequences that showed the best hits under the highest identity to one of the NR, KEGG, or Uniprot databases were retained. The candidate genes that have functions associated with luciferin were then selected. Moreover, literature search and manual curation were also performed to identify all possible putative enzymes involved in each step of the luciferin metabolic pathway.

### Validation of candidate gene expression using RT-PCR

To validate the expression of these candidate genes in the *L. aquatilis*, a different developmental stage, i.e., adult stage, which is also bioluminescent was selected for RT-PCR analysis. Total RNA was extracted from an adult *L. aquatilis* using the same method as previously described. The first strand DNA was amplified using RevertAid first strand cDNA (Thermo Scientific, USA). Reverse transcription reaction was performed using 2 μg of total RNA and oligo-dT primer as described in the user manual. PCR was then performed using Phire Hot Start II DNA Polymerase (Thermo Scientific, USA). The PCR reaction included 2 μL of the reverse transcriptase reaction mix, 1X Phire Reaction Buffer, 200 μM of each dNTPs, 0.5 μM of each primer (File S1), and Phire Hot Start II DNA Polymerase 1 Unit. The PCR reaction was performed under the following conditions: initial denaturation for 5 min at 98°C, 35 amplification cycles of denaturation for 40 s at 98°C, annealing for 30 s at 50°C, extension for 1 min at 72°C, and final extension for 5 min at 72°C. The amplified products were visualized by 1% agarose gel electrophoresis.

## Results and Discussion

### Transcriptome assembly of *L. aquatilis*

**Figure 1:**
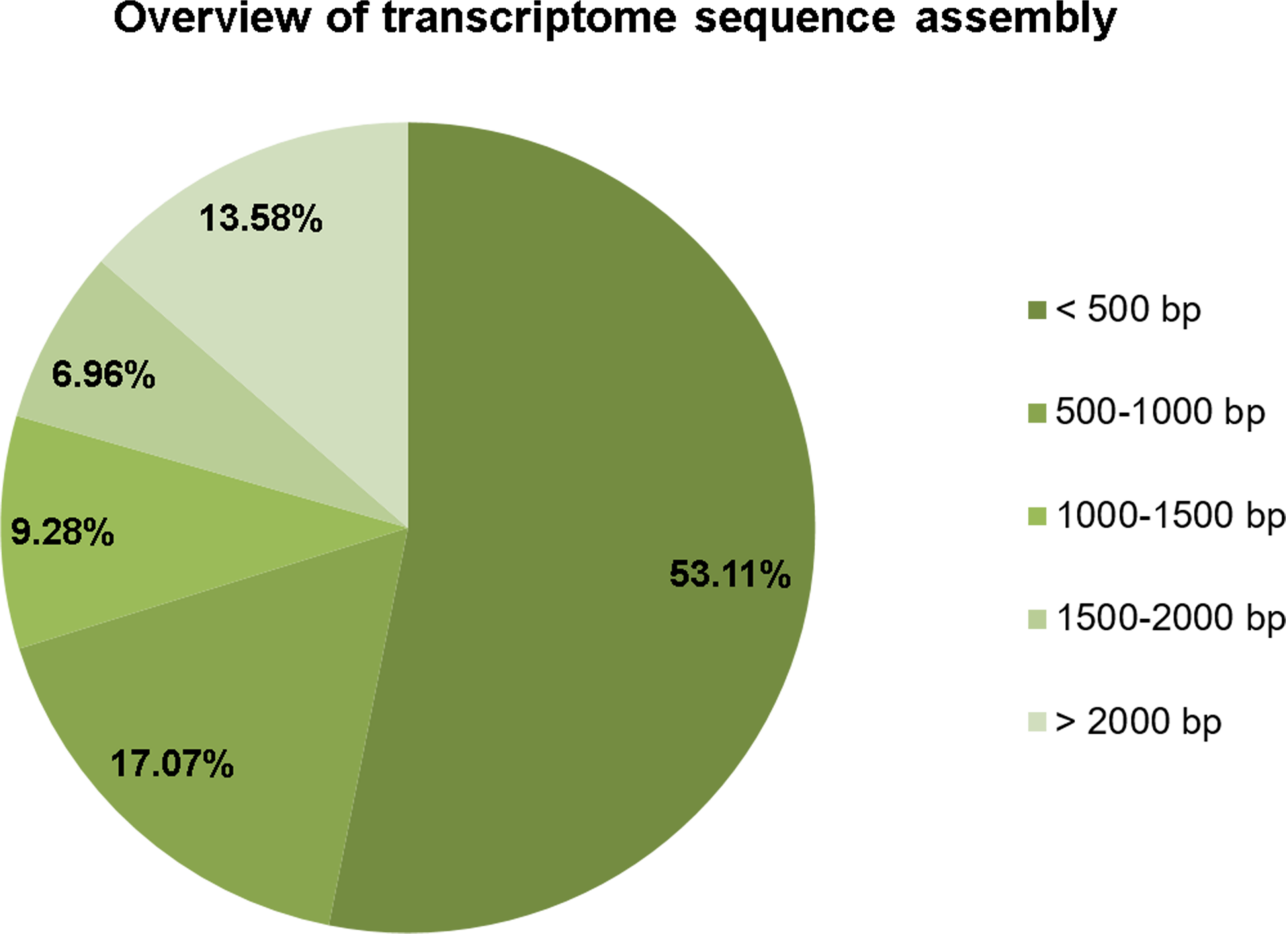
Size distribution of contigs. The size distribution of contigs demonstrated that the majority of the sequences was <500 bps.

The RNA extracted from the bioluminescent *L. aquatilis* larvae was sequenced using the Illumina HiSeq 2000 platform. A total of 63,533,268 raw reads with an average read length of 101 bp were obtained. After adapter trimming and removing of contaminating sequences and low-quality sequences, a total of 62,481,222 trimmed reads were retained. These trimmed reads were assembled into high-quality contigs with a total length of 38,873,002 bp and a total number of 39,730 contigs. The number of contigs without isoforms was 33,070. The maximum length of a contig was 26,786 bp, and the minimum length of a contig was 201 bp with an average length of 978.43 bp; an N50 of 1,889 bp were obtained (Table 1). The size distribution of contigs (Fig. 1) demonstrated that 21,102 contigs were <500 bp (53.11%), 6,780 contigs were 500–1,000 bp (17.07%), 3,686 contigs were 1,000–1,500 bp (9.28%), 2,767 contigs were 1,500–2,000 bp (6.96%), and 5,395 contigs were >2,000 bp (13.58%). The clean reads of *L. aqualtilis* larvae in this study were deposited in the NCBI SRA database under the accession number SRX1605859.

**Table 1:** Overview of the transcriptome. The transcriptome data of *L. aquatilis* larvae was obtained from Illumina HiSeq 2000 platform.

Info	All transcript contigs (only longest isoform per ‘GENE’)
Total raw reads	63,533,268
Total raw nucleotide	6,416,860,068
Total clean reads	62,481,222
Total clean nucleotides	6,228,699,940
Q20 percentage	99.20%
GC percentage	42.03%
Total trinity ‘genes’	39,730 (33,070)
Total trinity transcripts	39,730 (33,070)
Maximum length of contigs	26,786 (26,786)
Minimum length of contigs	201 (201)
Median length of contigs	451 (384)
Mean length of contigs	978.43 (833.32)
N50 of unigenes	1,889 (1,630)
Total assembled bases	38,873,002 (27,557,931)

### Functional annotation of *L. aquatilis*

**Figure 2:**
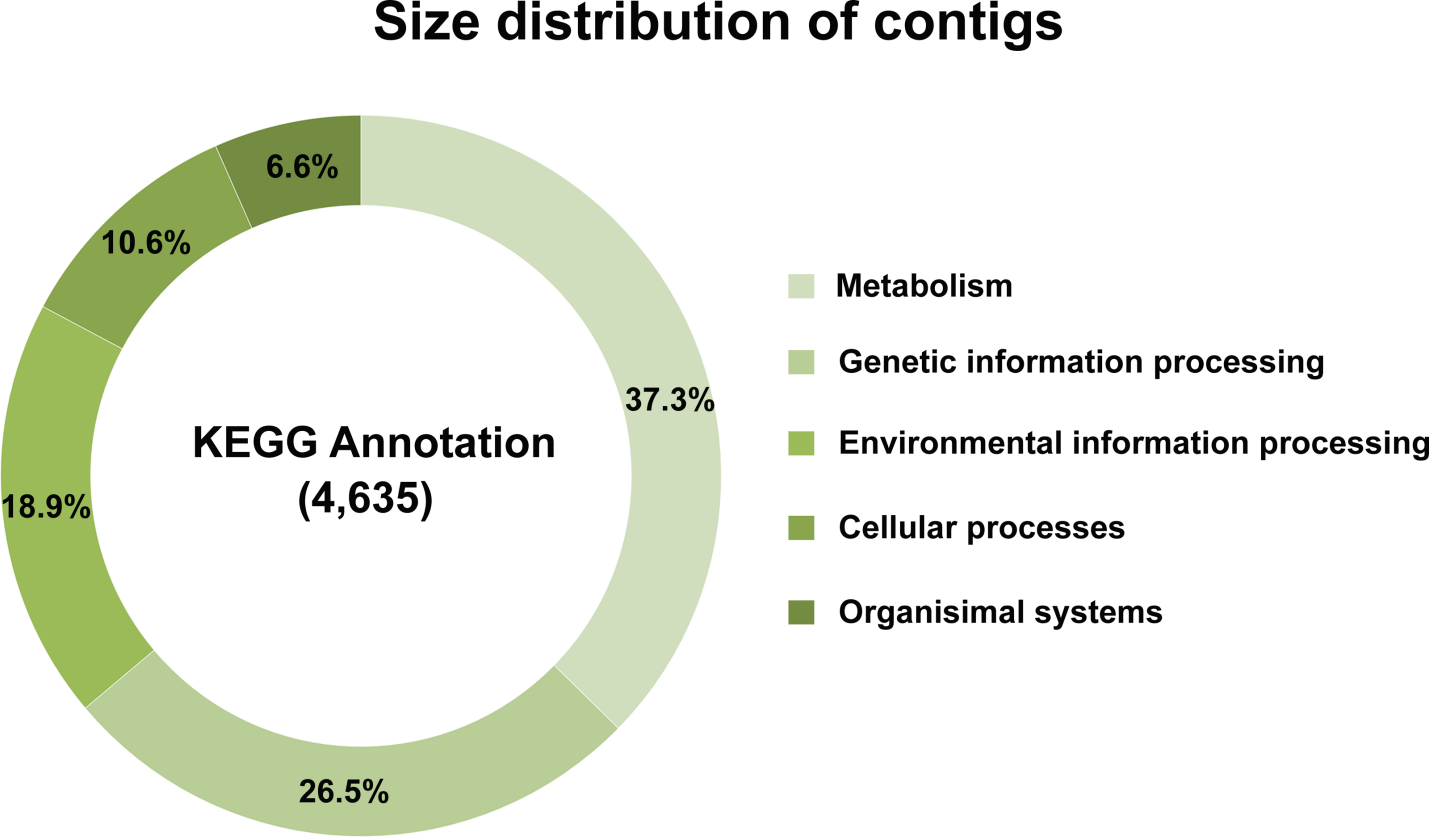
Overview of KEGG annotation. A total of 4,653 genes were grouped into five functional categories with the majority in the metabolism category.

**Figure 3:**
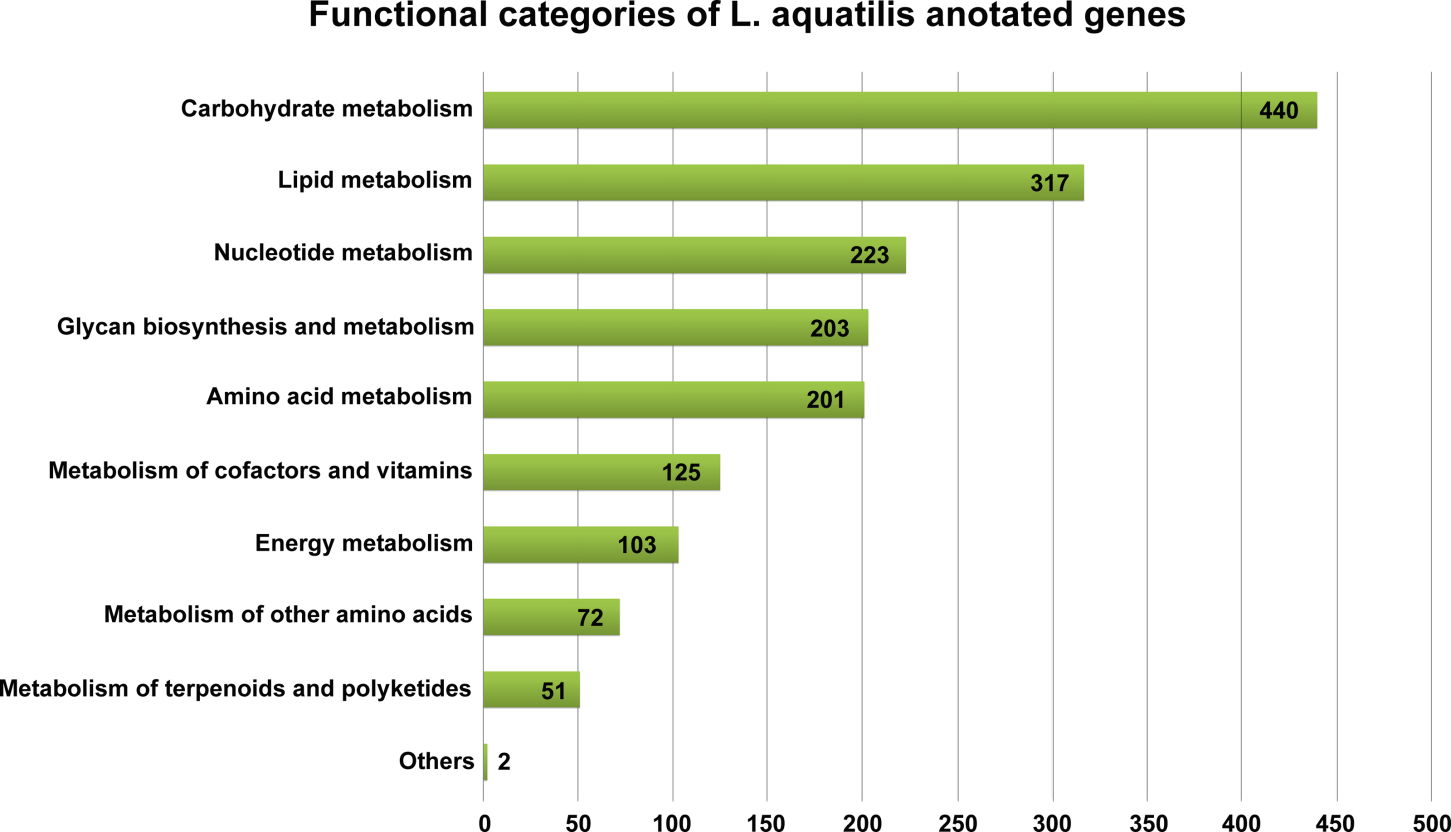
Metabolism functional categories of *L. aquatilis* annotated genes. The genes categorized in the metabolism category were further categorized into ten subcategories with the majority in the carbohydrate metabolism.

Of 39,730 assembled contigs, 19,761 protein-coding genes were identified using TransDecoder. These genes were subsequently annotated using three databases, KEGG, NR, and Uniprot, and the best match sequences from each of the databases were retained. A total of 14,025 (70.97%), 14,855 (75.17%), and 4,976 (25.18%) annotated protein-coding genes were obtained from NR, Uniprot, and KEGG, respectively (File S2). To further elucidate the functional and pathway association, the KEGG database was used (Kanehisa et al., 2011). Of the 4,976 KEGG annotated genes, a total of 4,653 genes were grouped into five functional categories, i.e., metabolism (1,737 genes, 37.3%), genetic information processing (1,233 genes, 26.5%), environmental information processing (881 genes, 18.9%), cellular processes (494 genes, 10.6%), and organismal systems (308 genes, 6.6%) (Fig. 2). From these results, the majority of predicted protein-coding genes from *L. aquatilis* were involved in metabolic functions. The genes in this functional category were further distributed into ten subcategories (Fig. 3). The genes associated in carbohydrate metabolism, lipid metabolism, and amino acid metabolism showed the highest numbers. This can be explained by the fact that genes in these three categories are involved in basic processes in living cells and are highly conserved and well-characterized pathways across the animal kingdom (Peregrín-Alvarez, Sanford & Parkinson, 2009). These results are consistent with transcriptome studies in other coleopteran insects, e.g., *Colaphellus bowringi* (Tan et al., 2015) and *Tomicus yunnanensis* (Zhu, Zhao & Yang, 2012). In contrast, the metabolism of the terpenoids and polyketides categories contained the least gene member, which is expected, as these secondary metabolites could be specific to each species. These compounds play several important roles in ecological interactions and also evolutionary aspects (Pankewitz & Hilker, 2008). Different groups of beetles have been reported to produce different defensive compounds. In the family Coccinellidae (ladybugs), many compounds, e.g., coccinelline from *Coccinella septempunctata* (Tursch et al., 1971), hippodamine from *Hippodamia convergens* (Tursch et al., 1972), and propyleine from *Propylaea quatuordecimpunctata* (Tursch, Daloze & Hootele, 1972) were identified. Both larva and adult leaf beetles (family Chrysomelidae) are also known to produce defensive compounds such as isoaxazolinone glucoside 5 and its 3-nitropropionate esters 6 to prevent them from natural enemies (Pauls et al., 2016). In the larvae of carabid beetle, *Chlaenius cordicollis*, various defensive compounds, e.g., methylhydroquinone, toluquinone 2,3-dimethylquinone were detected (Holliday, Mattingly & Holliday, 2015). Due to these diverse yet species-specific metabolites found among groups of insect, when the database searches were performed, only a small number of genes were annotated as being involved in the metabolism of terpenoids and polyketides in *L. aquatilis*.

**Figure 4:**
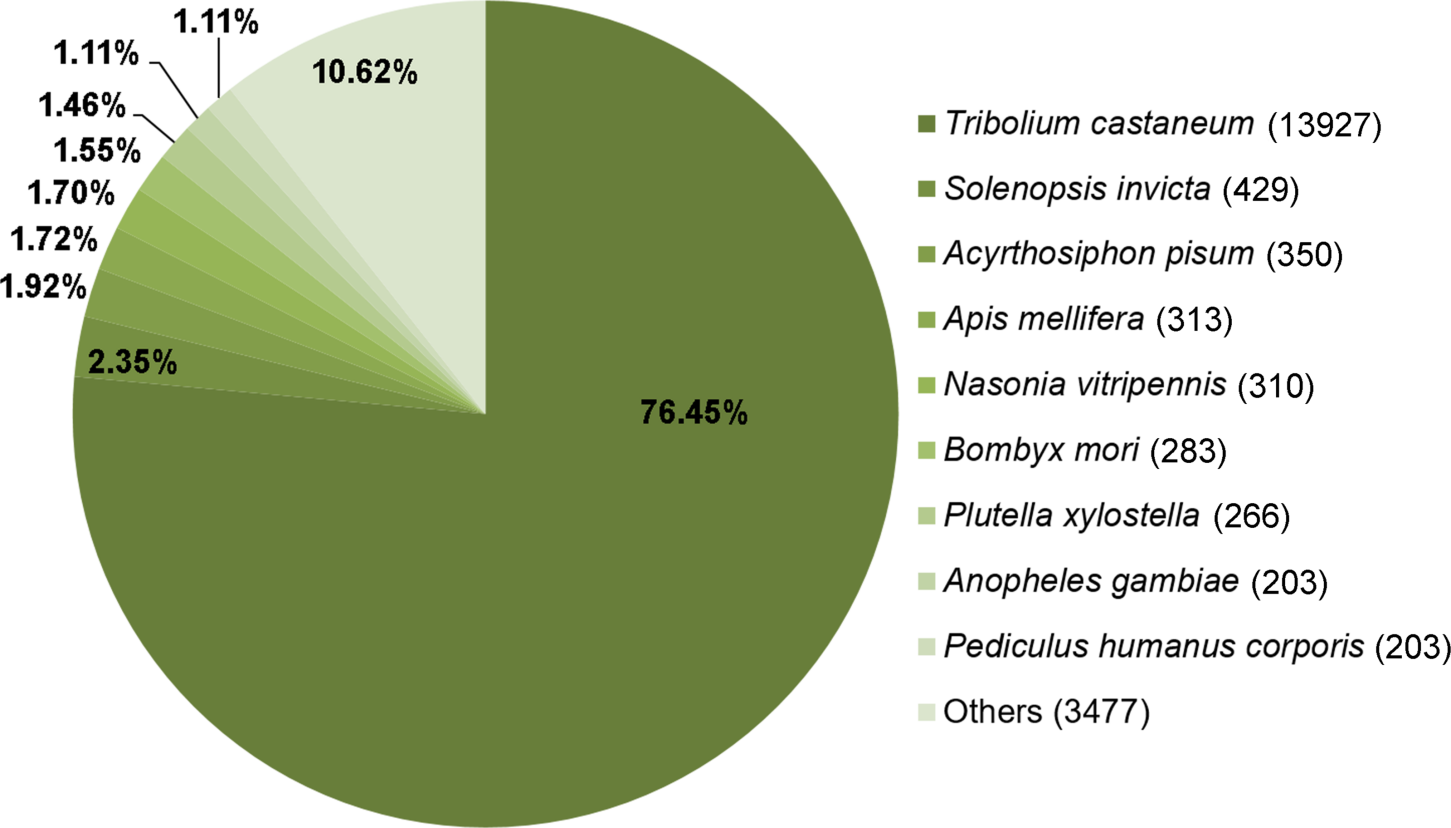
Comparative analysis of *L. aquatilis* KEGG-annotated genes with other arthropods. KEGG taxonomic mapping was performed using the 19,761 genes obtained from TransDecoder. The majority of these sequences matched the genes found in *Tribolium castaneum*.

To identify the orthologues of these annotated protein-coding genes in other taxa, KEGG taxonomic mapping was performed using the 19,761 genes obtained from TransDecoder. The majority of these sequences (>80%) matched the genes found in other arthropods. Of 13,927 genes, 70.48% observably matched the genes found in *Tribolium castaneum*, followed by *Solenopsis invicta* (429 sequences; 2.17%), *Acyrthosiphon pisum* (350 sequences; 1.77%), *Apis mellifera* (313 sequences; 1.58%), *Bombyx mori* (283 sequences; 1.43%), and the others (4,149 sequences; 21.01%) (Fig. 4). This result verifies the data obtained in this study as *T. casteneum* is the closest group of insect with whole genome data available for comparison. Therefore, it is not surprising that *L. aquatilis* would have the most orthologues with *T. castaneum*. Based on this, we selected to use the *T. castaneum* genome as the reference for further pathway mapping analysis.

### Comparative protein sequence analysis between *L. aquatilis* and *T. castaneum*

Based on KEGG taxonomic mapping, *T. castaneum* showed the highest homologues with *L. aquatilis* (76.45%). Although this *T. castaneum* is not bioluminescence, it is a model organism of Coleopteran insect, providing the most extensive list of proteins with which to compare; therefore, we selected this species for the comparative analysis. A comparative protein sequence analysis between these two species was then performed to identify conserved genes and their functions. The bidirectional best hits (BBH) analysis was performed between *L. aquatilis* (19,761 genes) and *T. castaneum* (18,076 genes). As a result, a total of 8,020 conserved genes were identified (File S3). These conserved genes are mostly involved in common biological pathways found in most insects, such as growth and development, lipid metabolism, and energy metabolism, e.g., juvenile hormone epoxide hydrolase, fatty acid hydroxylase, and V-type proton ATPase, respectively (File S3).

**Figure 5:**
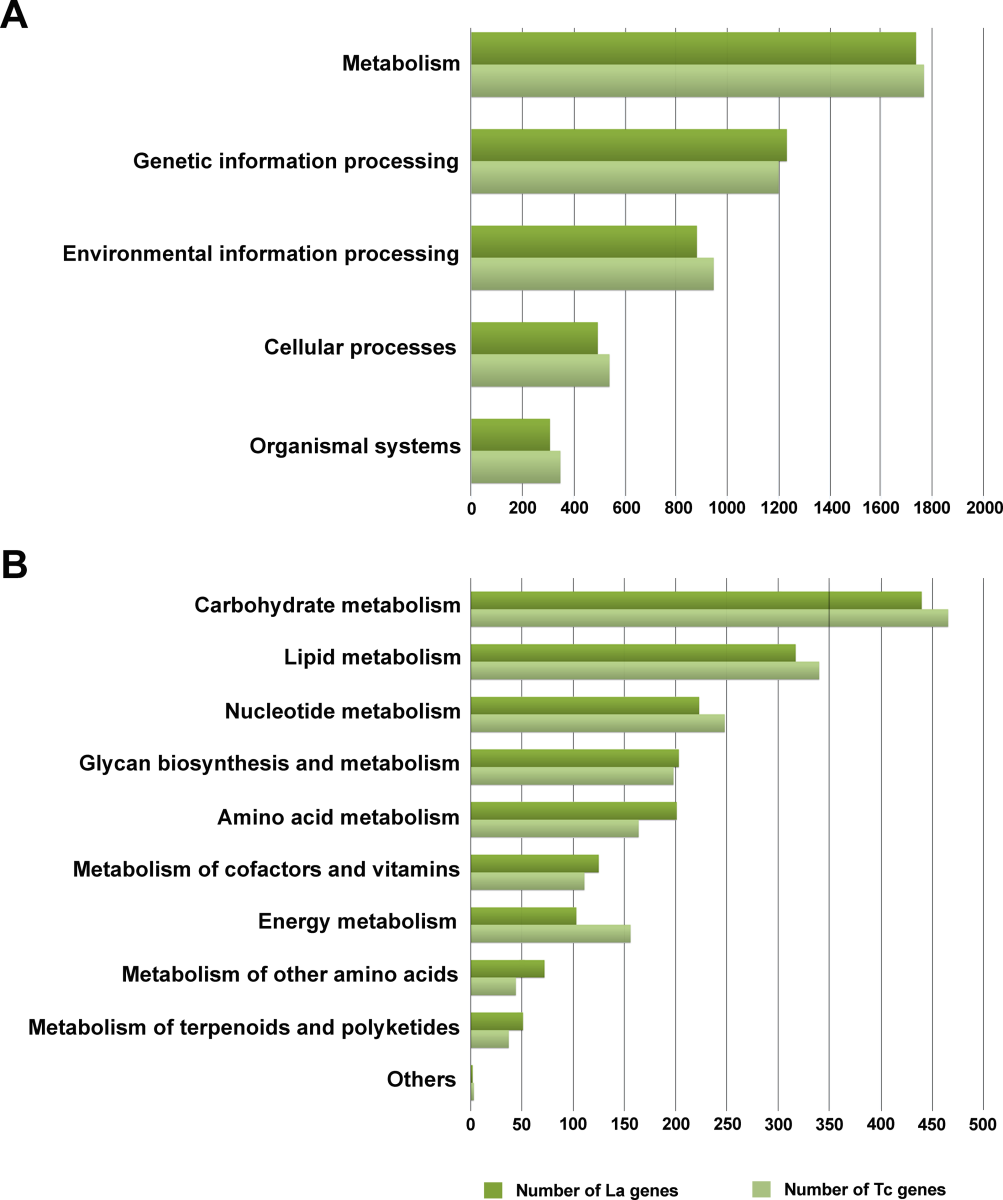
Comparative protein sequence analysis between *L. aquatilis* and *T. casteneum.* A pairwise comparison of putative gene sequences between *L. aquatilis* and *T. castaneum* demonstrated a similar functional distribution trend, with the highest number of genes in the metabolism category (A). The genes in this metabolism category were further compared in the sub-category level (B).

Assessing gene functional distribution in KEGG between *L. aquatilis* (4,653 genes) and *T. castaneum* (4,803 genes), similar trends were found as illustrated in Fig. 5, the highest number of genes was found in the metabolism category. Interestingly, the numbers of genes in energy metabolism, nucleotide metabolism, lipid metabolism, and carbohydrate metabolism were higher in *T. castaneum* than in *L. aquatilis.* In contrast, the numbers of genes in the metabolism of cofactors and vitamins, amino acid metabolism, metabolism of terpenoids and polyketides, metabolism of other amino acids, and glycan biosynthesis and metabolism were higher in *L. aquatilis* than in *T. castaneum*. It is worth noting that our transcriptome data was obtained only from the larvae of the firefly, but the data set from *T. castaneum* included annotated protein data from the genome that represents the proteins in both adult and larval stages (ftp://ftp.ncbi.nih.gov/genomes/Tribolium_castaneum/protein/). Not surprising, the genes involved in energy metabolism are likely to be higher in number in *T. castaneum*. In many insects, the processes during larval stages are mainly involved in food and energy storage for intense growth, while reproduction tends to be the main focus during the adult stage (Arnold, Cassey & White, 2016). These results coincided with feeding behaviors of both species. *T. castaneum* has an extremely carbohydrate-rich diet. It is considered a pest of storage grains and cereal products (Perkin, Elpidina & Oppert, 2016). In contrast, the larvae of *L. aquatilis* have a protein-rich diet as they consume only aquatic snails during this larval stage (Thancharoen, Ballantyne & Branham, 2007). On the contrary, genes in the metabolism of terpenoids and polyketides were higher in the *L. aquatilis* than in the *T. castaneum*, suggesting the complexity of the terpenoids and polyketides pathways in the firefly. Apart from the juvenile hormones that are present in both *T. castaneum* and *L. aquatilis*, not many other terpenoids and polyketides were reported in the *T. castaneum* eventhough it is a model beetle, and the genes have been well characterized. Several quinone derivatives were reported to be defensive compounds in the *T. castaneum* (Unruh, Xu & Kramer, 1998; Villaverde, Juárez & Mijailovsky, 2007). Another compound of terpenoids and polyketides found in *T. castaneum* was 4,8-Dimethyldecanal (4,8-DMD), an aggregation pheromone produced by male *T. castaneum* (Kim, Matsuyama & Suzuki, 2005). In fireflies, defensive steroidal pyrones (lucibufagins) released via a defensive behavior called reflexed-bleeding were detected in many genera including *Photinus, Ellychnia,* and *Lampyris* (González, Hare & Eisner, 1999; Tyler et al., 2008; Mosey et al., 2015). Another compound, a defensive betaine, N-methylquinolinium 2-carboxylate, was found in the adults and female larvae of *Photuris* (González *et al.*, 1999; Trice, Tyler & Day, 2004). Furthermore, over 10 novel steroids from *Lucidota atra* were detected using capillary NMR spectroscopy (Gronquist et al., 2005). Although a direct relationship between these defensive compounds and bioluminescence reaction has never been reported, bioluminescence in the firefly is often used for defensive signaling to warn predators of its non-palatability and toxicity (Trice, Tyler & Day, 2004; Fu et al., 2007).

### Identification of candidate genes involved in luciferin metabolic pathway

**Figure 6:**
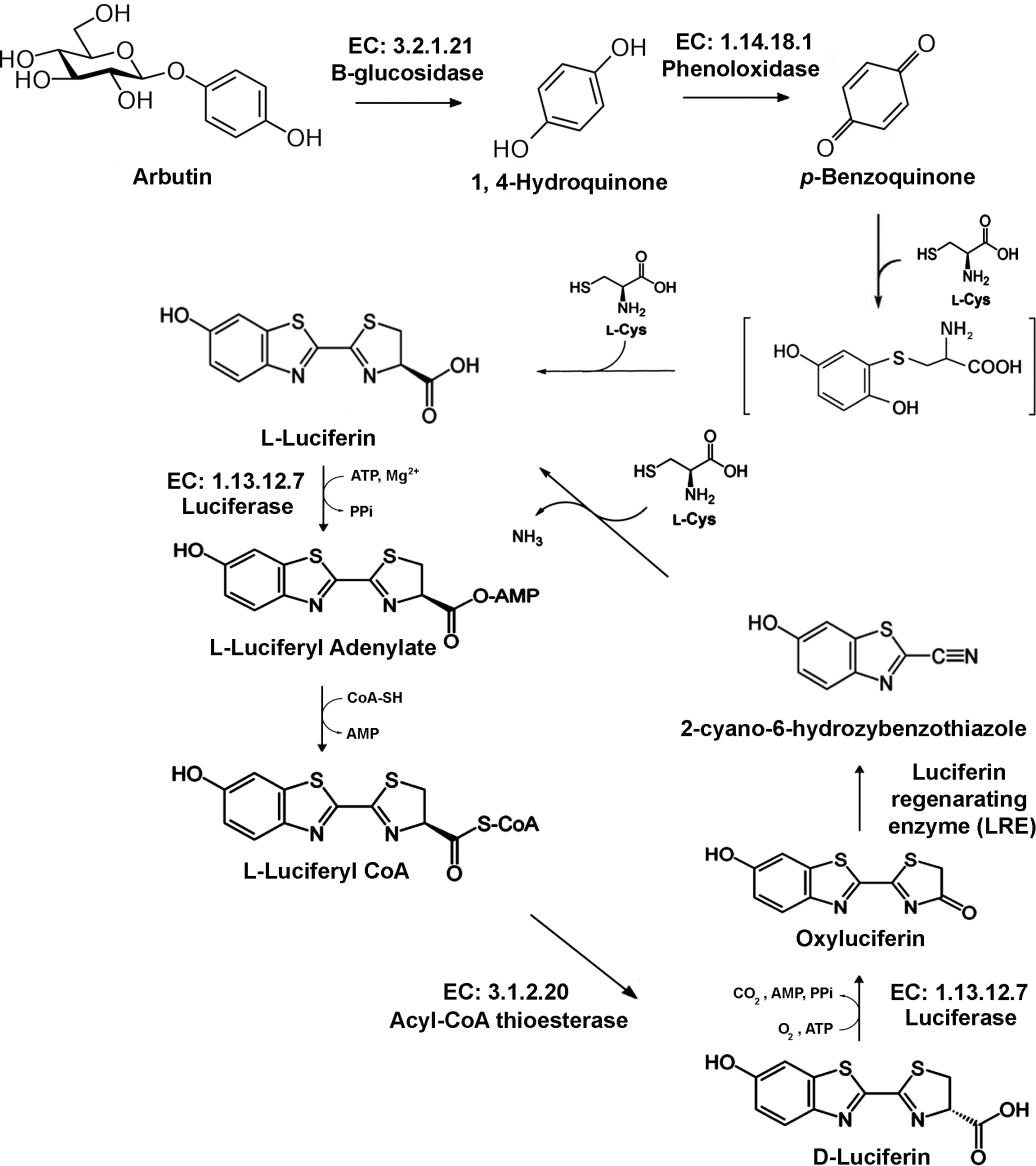
Proposed luciferin metabolic pathway. A total of 11 candidate genes involved in the luciferin metabolic pathway were identified in the *L. aquatilis* transcriptome data (adapted from Niwa, Nakamura & Ohmiya, 2006; Oba et al., 2013; Hemmati et al., 2015; Kanie et al., 2016).

A total of 11 candidate genes involved in the luciferin metabolic pathway were identified in the *L. aquatilis* transcriptome (Table 2 and Fig. 6). These genes were selected from protein sequences that matched one of the NR, KEGG, or Uniprot databases. These 11 genes, were annotated as putative *β*-glucosidase enzymes (EC: 3.2.1.21), phenoloxidase (EC: 1.14.18.1), luciferase (EC: 1.13.12.7), thioesterase (EC: 3.1.2.20), and luciferin regenerating enzyme (LRE). Most of the candidates obtained for this study used integration of NR and Uniprot, whereas none of the candidates were identified using KEGG. This result indicated that the KEGG database contains limited information about protein functions associated with bioluminescence, whether for fireflies or any other bioluminescent insects (Kanehisa et al., 2016), in comparison with the other two databases.

**Table 2:** List of candidate enzymes involved in the luciferin metabolic pathway. A total of 11 candidate genes involved in the luciferin metabolic pathway were identified in the *L. aquatilis* transcriptome data. An elongation factor 1-alpha was also identified to be used as a control in the RT-PCR experiment.

EC number	Protein name	Transcript ID	FPKM	Protein ID	Functional description	Accession	**Database**			Amino acid sequence identity (%)	E-value
											
							NR	KEGG	Uniprot		
**EC: 3.2.1.21**	B-glucosidases	c14185_g1_i1	10.85	c14185_g1_i1| m.15351	Aryl-phospho-beta-D-glucosidase BglA [*Bacillus subtilis* (strain 168)]	Q17PP1	✓^a^		✓	44.95	2E–58
		c11559_g1_i1	1.3	c11559_g1_i1| m.9194	Cytosolic beta-glucosidase [*Cavia porcellus*]	P97265	✓^a^		✓	41.99	9E–99
**EC:1.14.18.1**	Phenol oxidases	c14353_g1_i1	230.43	c14353_g1_i1| m.15909	Phenoloxidase 2 [*Drosophila melanogaster*]	Q9V521	✓^a^		✓	57.37	0
		c14376_g1_i1	320.14	c14376_g1_i1| m.15966	Phenoloxidase 2 [*Drosophila melanogaster*]	Q9V521	✓		✓	62.26	0
**EC: 1.13.12.7**	Luciferases	c10041_g1_i1	112.72	c10041_g1_i1| m.6619	Luciferase [*Luciola lateralis*]	Q01158.1	✓		✓	83.67	0
		c13054_g1_i1	65.83	c13054_g1_i1| m.12283	Firefly luciferase [*Luciola cruciata*]	BAJ41368.1	✓		✓	79.04	0
**EC: 3.1.2.20**	Thioesterases	c9513_g1_i1	9.32	c9513_g1_i1| m.5804	Acyl-coenzyme A thioesterase 13 [*Saimiri boliviensis boliviensis*]	XP_003927347.1	✓		✓^a^	87.06	0
		c13177_g1_i1	9.7	c13177_g1_i1| m.12638	Acyl-coenzyme A thioesterase 10, mitochondrial [*Tribolium castaneum*]	XP_973760.2	✓		✓^a^	60.86	7E–179
**N/A**	Luciferin regenerating enzyme (LRE)	c10156_g1_i1	55.04	c10156_g1_i1| m.6798	Luciferin regenerating enzyme [*Lampyris turkestanicus*]	ADK55065.1	✓		✓^a^	78.18	0
		c12106_g1_i1	24.61	c12106_g1_i1| m.10324	Luciferin-regenerating enzyme [*Luciola cruciata*]	BAB85479.1	✓		✓^a^	74.43	2E–170
		c8279_g1_i1	0.68	c8279_g1_i1| m.4268	Luciferin-regenerating enzyme [*Luciola cruciata*]	BAB85479.1	✓		✓^a^	56.11	4E–43
**N/A**	Elongation factor 1-alpha	c11516_g1_i1	4.3	c11516_g1_i1| m.9114	Elongation factor 1-alpha [*Tribolium castaneum*]	XP_966355.1	✓	✓	✓	94.24	0

**Notes.**

a Predicted gene name in the database differs from what is presented in the subject column (File S2).

### Proposed luciferin metabolism of *L. aquatilis*

The luciferin biosynthesis is thought to be generated from 1,4-hydroquinone (Oba et al., 2013; Fig. 6). This 1,4-hydroquinone is proposed to be stored as arbutin in the firefly lantern. Only arbutin, but not 1,4-hydroquinone, was detected in the adult firefly lantern by HPLC, suggesting that 1,4-hydroquinone was immediately oxidized to 1,4-benzoquinone to produce luciferin (Oba et al., 2013). This 1,4-hydroquinone is hydrolyzed from arbutin by arbutin hydrolysis enzymes, i.e., glucosidases (Reinhard et al., 2002; Oba et al., 2013). In this study, we identified two putative *β*-glucosidase enzymes, c14185 (FPKM: 10.85) and c11559 (FPKM: 1.3) from the transcriptome data. However, hydroquinone has other functions in the developmental pathway of arthropods as it can also be used to crosslink proteins in the cuticle (Shear, 2015). This suggests the role of hydroquinone in *L. aquatilis* larvae could be as a precursor of both the luciferin pathway and cuticle production.

The next step in luciferin metabolism is an oxidation of 1,4-hydroquinone into 1,4-benzoquinone (Fig. 6). The incorporation efficiency of the [D_4_]-benzoquinone into firefly luciferin was higher than that of [D_6_]-hydroquinone, indicating that 1,4-hydroquinone may convert to 1,4-benzoquinone in the biosynthesis of luciferin in the firefly lantern (Oba et al., 2013). The transcriptome analysis of the *T. castaneum* odoriferous defensive stink gland revealed candidate enzymes, i.e., glucosidases, phenol oxidases, and peroxidases, that are involved in the production of quinones (Li et al., 2013). The substrate (1, 4-hydroquinone) and the product (p-benzoquinone) were searched in the *T. castaneum* KEGG pathway. The pathway that was identified to convert 1,4-hydroquinone into p-benzoquinone was tca00740 (riboflavin metabolism). In fireflies, p-benzoquinone is a precursor for luciferin biosynthesis (Oba et al., 2013) and it could possibly be synthesized using phenol oxidases. In this study, two candidate genes encoding for phenol oxidases, c14353 (FPKM: 230.43) and c14376 (FPKM: 320.14), were identified. Benzoquinones are toxic compounds reported to be produced in many arthropods including beetles as a defensive mechanism (Ibarra & Blair, 2013; Rocha et al., 2013). In cockchafer, benzoquinone is used to attract mates and protect the larvae from pathogenic bacteria and fungi (Ruther et al., 2001; Ruther, Podsiadlowski & Hilker, 2001). However, the defensive compounds in fireflies were reported to be steroid pyrones called lucibufagins (Eisner et al., 1978; Meinwald, Wiemer & Eisner, 1979; Eisner et al., 1997) and betaine N-methylquinolinium 2-carboxylate (González et al., 1999). The lucibufagins are produced by the adult of the North American firefly genus *Photinus* (Eisner et al., 1978; Eisner et al., 1997). Interestingly, the adult fireflies of the genus *Photuris* acquire these compounds from *Photinus* fireflies through consumption (Eisner et al., 1997; Faust, De Cock & Lewis, 2012). These defensive compounds are released when the fireflies are disturbed through the chemical defensive behavior called “reflexed-bleeding” (Blum & Sannasi, 1974; Eisner et al., 1997). This behavior is also observed in other firefly genera, e.g., *Pyrocoelia* (Wang et al., 2007) and *Asymmetricata* (A Sriboonlert, pers. obs., 2013). In the larvae of many genera including *Lampyris, Luciola,* and *Nyctophila*, pleural defensive organs have been identified which secrete a repellent substance used as a defensive mechanism (Trice, Tyler & Day, 2004). In aquatic firefly larvae, *Luciola leii*, a closely related species of *L. aquatilis* it is reported to produce two volatile terpenoids: terpinolene and *γ*-terpinene from thoracic and abdominal glands as repellent compounds (Fu et al., 2006b; Fu et al., 2007). From these findings, benzoquinone is unlikely to be used directly in fireflies as a defensive compound, and instead is likely solely used for the production of luciferin. Nonetheless, it is possible that these quinone substances could also have indirect benefits as defensive compounds against pathogenic bacteria and predators.

After p-benzoquinone is obtained, it is converted into L-luciferin in the presence of L- cysteine (Fig. 6). This reaction has been proven to occur nonenzymatically (Kanie et al., 2016). L-luciferin is demonstrated to be generated from p-benzoquinone and cysteine in various neutral buffers without any enzymes (Kanie et al., 2016). Nonetheless, L-luciferin was demonstrated to act as a D-luciferin antagonist in bioluminescence (Lembert, 1996; Niwa, Nakamura & Ohmiya, 2006; Nakamura et al., 2005). The chirality of luciferin is also important in the bioluminescence reaction (Niwa, Nakamura & Ohmiya, 2006). In the firefly lantern, L-luciferin was produced from L-cysteine. However, the substrate for luciferase in firefly bioluminescence was reported to be D-luciferin, whereas L-luciferin acts as an inhibitor of the bioluminescence reaction (Seliger et al., 1961; Lembert, 1996; Niwa, Nakamura & Ohmiya, 2006; Inouye, 2010). Endogenous luciferin from the adult fireflies was detected in both D- and L-forms, with more of the D-form than the L-form (Niwa, Nakamura & Ohmiya, 2006; Oba et al., 2013). With evidence from the incorporation study and the measurement of D- and L-luciferin levels in different developmental stages of firefly, this suggests that that the L-luciferin is a biosynthetic intermediate of D-luciferin (Niwa, Nakamura & Ohmiya, 2006; Oba et al., 2013). The conversion of L-luciferin to D-luciferin is demonstrated to be an enzymatic reaction (Niwa, Nakamura & Ohmiya, 2006) with ATP, Mg^2+^, and CoA suggesting the function of CoA-thioesterase hydrolysis of D-luciferyl CoA to yield D-luciferin (Niwa, Nakamura & Ohmiya, 2006; Hunt et al., 2006; Niwa, Nakamura & Ohmiya, 2006; Niwa, Nakajima & Ohmiya, 2010). However, Inouye (2010) suggest the racemization between D-LH2-AMP and L-LH2 AMP might not occur in the luciferase molecule, but in the solution non-enzymatically after releasing luciferyl, forming adenylate from the luciferase molecule. In this study, two acyl-CoA thioesterases, c9513 (FPKM: 9.32) and c13177 (FPKM: 9.7), corresponding to EC: 3.1.2.20 were identified. Relatively low expression of this enzyme could be due to the low accumulation of D-luciferin at the larval stage. In *L. cruciata*, D-luciferin was detected at the highest concentration in the adult stage (Niwa, Nakamura & Ohmiya, 2006).

The next step of the luciferin metabolic pathway is where the actual bioluminescence occurs (Fig. 6). Luciferase is crucial for the bioluminescence reaction. Firefly luciferase (EC. 1.13.12.7) oxidizes the luciferin substrate with the presence of cofactors Mg^2+^, O_2_, and ATP to produce oxyluciferin and emit yellow-green light (Inouye, 2010). In this study, two candidate luciferases, c10041 (FPKM: 112.72) and c13054 (FPKM: 65.83), were identified. One of these luciferases, c10041, demonstrated the highest FPKM of 112.72 and has the highest homology to *L. lateralis* luciferase, with 83.67% identity. Another luciferase enzyme, c13054, with FPKM of 65.83, also has high homology to the *L. cruciata* luciferase (79.04%). In addition, five putative luciferases were identified in this study, i.e., c12163 (FPKM: 17), c14996 (FPKM: 5.31), c18617 (FPKM: 1.09), c13390 (FPKM: 3.73), and c14833 (FPKM: 5.58) (File S2), with much lower identity scores (<45%) and FPKM. These luciferases may have other functions not involving bioluminescence. Previous studies report only one luciferase enzyme in each species that is responsible for the bioluminescence reaction (Viviani et al., 2004). However, a recent study demonstrates a luciferase isotype LcLuc2 in *Luciola cruciata* (Oba et al., 2010). Both LcLuc1 and LcLuc2 show luminescence activity and fatty acyl-CoA synthetic activity (Tsutomu, Hiroki & Eiichi, 1989; Oba et al., 2010). In addition, luciferase paralogs (LcLL1 and LcLL2) are also identified in the same species (Oba et al., 2006). However, neither the LcLL1 nor LcLL2 show enzymatic activity (Oba et al., 2006). Apart from the bioluminescence reaction, luciferase is found to catalyze fatty acyl-CoA synthesis from fatty acids in the presence of ATP, Mg^2+^, and CoA. The luciferase enzyme is hypothesized to have evolved from fatty acyl-CoA synthetase (Inouye, 2010). Moreover, the fatty acyl-CoA synthetase enzyme from non-luminous insects can be converted into luciferase by site-directed mutagenesis (Inouye, 2010), suggesting the other luciferase candidates identified in this study may actually be fatty acyl-CoA synthetase enzyme.

The last step in the luciferin metabolic pathway is the recycling of oxyluciferin. Oxyluciferin is reported to be recycled into 2-cyano-6-hydrobenzothiazole (CHBT) by luciferin regenerating enzyme (LRE; Fig. 6) (Gomi & Kajiyama, 2001; Gomi, Hirokawa & Kajiyama, 2002; Day, Tisi & Bailey, 2004; Niwa, Nakamura & Ohmiya, 2006; Marques & da Silva, 2009; Emamzadeh et al., 2010; Inouye, 2010; Hemmati et al., 2015). The CHBT then combines with L-cysteine and is converted into L-luciferin (Fig. 6). This step is reported to occur without enzymes (Okada et al., 1974; Gomi & Kajiyama, 2001; Day, Tisi & Bailey, 2004). Incorporation experiments demonstrate incorporations of 2-cyano-6-hydrobenzothiazole (CHBT), and D- and L- cysteine into D-luciferin in fireflies (McCapra & Razavi, 1976; Okada, Iio & Goto, 1976; Day, Tisi & Bailey, 2004; Niwa, Nakamura & Ohmiya, 2006). Both D- and L- forms of luciferin are found in the firefly at a different ratio at different stages of development (Lembert, 1996; Niwa, Nakamura & Ohmiya, 2006). In the presence of LRE and D-cysteine, an increase of bioluminescence is observed, but in the absence of LRE, bioluminescence only appears for nine minutes (Gomi & Kajiyama, 2001; Gomi, Hirokawa & Kajiyama, 2002; Hemmati et al., 2015). In this study, we identified three putative LREs, c10156 (FPKM: 55.04), c12106 (FPKM: 24.61), and c8279 (FPKM: 0.68). The c10156 DNA sequence was very similar to the *T-LRE* from *Lampyris turkestanicus* (Alipour et al., 2004), with 78.18% amino acid sequence identity. The other two LREs, c12106 and c8279, show highest similarity to the *LRE* from *Luciola cruciata* (Gomi, Hirokawa & Kajiyama, 2002) with 74.43% and 56.11% amino acid sequence identity, respectively. This is the first report of multiple transcripts of LRE identified in one species of firefly. However, the role of these three LREs in *L. aquatilis* remains to be confirmed.

### Validation of candidate gene expression using RT-PCR

**Figure 7:**
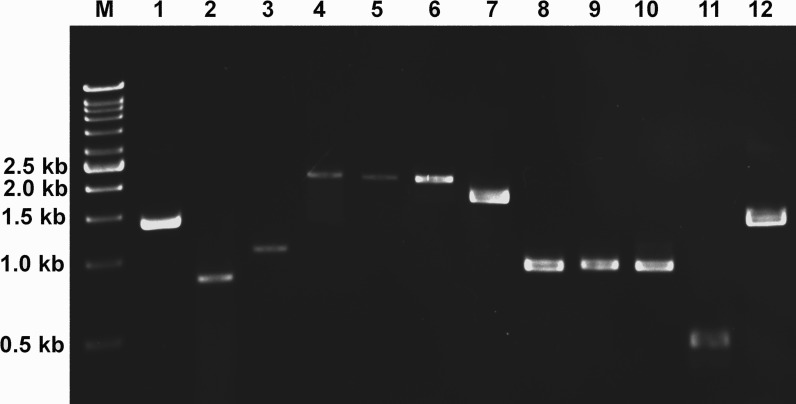
Gene expression analysis of candidate genes using reverse transcription PCR (RT-PCR). Expression of candidate genes in luciferin metabolic pathway was analyzed by RT-PCR. Elongation factor 1-alpha (lane 1) was used as a control. M: DNA ladder (1 Kb), 1: c11516_g1_i1 (*Elongation factor 1-alpha*, 1,383 bp), 2: c14185_g1_i1 (*B-glucosidase*, 819 bp), 3: c11559_g1_i1 (*B-glucosidase*, 1,049 bp), 4: c14353_g1_i1 (*Phenoloxidase*, 2,058 bp), 5: c14376_g1_i1 (*Phenoloxidase*, 2,046 bp), 6: c13054_g1_i1 (*Luciferase*, 2,022 bp), 7: c10041_g1_i1 (*Luciferase*, 1,635 bp), 8: c10156_g1_i1 (*Luciferin regenerating enzyme*, 921 bp), 9: c12106_g1_i1 (*Luciferin regenerating enzyme*, 930 bp), 10: c8279_g1_i1 (*Luciferin regenerating enzyme*, 927 bp), 11: c9513_g1_i1 (*Thioesterase*, 450 bp), 12: c13177_g1_i1 (*Thioesterase*, 1,374 bp).

The expression of 11 candidate genes identified in this study was verified using RT-PCR (Fig. 7). This validation was performed using a different developmental stage from the RNA-seq experiment to confirm the expression of these candidate genes in *L. aquatilis*. All candidate genes involved in the luciferin metabolic pathway are expected to be expressed in both larval and adult stages as fireflies at these two developmental stages are bioluminescence. Of seven candidate luciferase genes, only the two with the highest identity with known firefly luciferases were selected for RT-PCR analysis. An elongation factor 1-alpha (*EF1*α**) gene (c11516; FPKM: 4.3) was selected as an internal control because it was presented in all three databases. It is not a common reference gene, but it was used because it is one of the most stable reference genes in another coleopteran, *C. bowringi* (Tan et al., 2015). On the other hand, more common reference genes, e.g., *tubulin* and *GAPDH*, were shown to be less stable (Tan et al., 2015). The results show that the 11 candidate genes are expressed, verifying the transcriptome data analyzed in this study. Nonetheless, further experiments e.g., gene knockout and gene expression analysis are required to prove the function of these candidate genes in each step of luciferin biosynthesis.

## Conclusions

In this study, we identify candidate genes involved in the firefly bioluminescence reaction from transcriptome data of bioluminescent *L. aquatilis* larvae. Here, we proposed a list of enzymes involved in the firefly luciferin metabolic pathway. The expression of these enzyme-encoding genes is demonstrated in the adult stage of the firefly to confirm our transcriptome results. Although candidate enzymes of the luciferin biosynthetic pathway have been identified in this study, the actual function of these enzymes still need to be verified. Gene knockout will be performed in the fireflies to confirm the functions of these candidate genes by using the recent genome editing technology. Moreover, gene expression analyses will also be performed to confirm the involvement of these enzymes in the luciferin metabolic pathway. By elucidating the luciferin biosynthetic pathway, the development of firefly bioluminescence applications will be extended. Currently, applications using the firefly bioluminescence reporter system primarily rely on commercially synthesized luciferin to generate luminescence. Many applications can benefit from the elucidation of luciferin biosynthesis. Autoluminescence of modified organisms is ideal for this reporter system. Other bioluminescence systems, e.g., bacterial lux that can be seen without any light sources, have also been used in similar applications. Recently, a group of researchers generated an autoluminescent plant from a bacterial lux system (Krichevsky et al., 2010). However, one of the main problems of the bacterial lux system is the bioluminescence intensity especially in the *in vivo* bioluminescence assay (Mudge, Lewis-Henderson & Birch, 1996). In contrast, firefly LUC system was proven to be more efficient detectable by luminometer assays in the intact tissues of plants (Mudge, Lewis-Henderson & Birch, 1996). Prior attempts to create firefly bioluminescent plants were first examined in 1986 (Ow et al., 1986), but as the luciferin biosynthesis process was still mysterious, the plants could only glow in contact with applied luciferin substrate. By understanding firefly luciferin metabolic pathways, we can develop a self-sustainable system without having to constantly apply the luciferin substrate. This also has potential to be used in live cell imaging and related technologies in the future.

##  Supplemental Information

10.7717/peerj.2534/supp-1 File S1 Primers used in reverse transcription PCR (RT-PCR) analysis

10.7717/peerj.2534/supp-2 File S2 Annotated protein-coding genes via NR, Uniprot, and KEGG

10.7717/peerj.2534/supp-3 File S3 Bidirectional hits of candidate genes between *T. castaneum* and *L. aquatilis.*

10.7717/peerj.2534/supp-4 File S4 Amino acid sequences

## Additional Information and Declarations

### DNA Deposition

The following information was supplied regarding the deposition of DNA sequences: GenBank (SRX1605859).

### Data Availability

The following information was supplied regarding data availability: The raw data has been supplied as Supplementary File.
